# The changes in the nursing practice environment brought by COVID-19 and improvement recommendations from the nurses’ perspective: a cross-sectional study

**DOI:** 10.1186/s12913-022-08135-7

**Published:** 2022-06-06

**Authors:** Cheng Jingxia, Zhu Longling, Zuo Qiantao, Peng Weixue, Jiang Xiaolian

**Affiliations:** West China School of Nursing, Sichuan University/West China Hospital, Sichuan University, Chengdu, China

**Keywords:** COVID-19, Nurse, Practice environment, Organization and administration, Working condition

## Abstract

**Background:**

The COVID-19 pandemic has brought an opportunity to increase investment in the nursing practice environment, which has greatly impacted patients, nurses, and organizations. However, there were limited studies concerning the changes in the practice environment since the COVID-19 pandemic and the way to improve it from nurses’ perspectives.

**Methods:**

A cross-sectional study was conducted among 460 nurses from seven hospitals in Sichuan, China. Both the quantitative and qualitative data were collected from an online questionnaire. The quantitative data were collected using the Chinese version of the Practice Environment Scale-Nursing Work Index and compared with available norms in 2010. The qualitative data were collected through an open question following the scale and analyzed by content analysis.

**Results:**

The mean of the score of the practice environment scale was 3.44 (SD = 0.56) out of 4.00. The score of the total scale and the dimensions were significantly higher than the norms, apart from nurse-physician relations and nurse participation in hospital affairs. The qualitative findings revealed positive changes in nursing foundations for quality of care, nurse participation in hospital affairs and nurse-physician relations, and poor staffing and resource adequacy. The improvement in the working model and ward environment is the primary concern of nurses.

**Conclusions:**

The COVID-19 pandemic brought some positive changes in the nursing practice environment, but more efforts are needed to solve those nagging and important problems, such as staff shortages and low participation. Nursing managers and hospital leaders were encouraged to listen to nurses’ concerns and value this suitable opportunity for changing and improving to achieve better health services and coping ability to deal with emergency events going forward. Improving the ward environment and taking a professional model instead of sticking to the tedious process might be worthwhile.

## Background

The global pandemic of COVID-19 placed many challenges on nursing and a stark reminder of nurses’ vital roles in health care [1, 2]. Long-term efforts are still needed to overcome the pandemic for the foreseeable future completely. Thus, it is important to increase investment in nursing to reduce barriers to nurses’ work and promote their work enthusiasm.

As an essential factor of nurses’ work, the nursing practice environment, defined as the organizational characteristics of a work setting that enhance or constrain professional nursing practice [3], is worth attention for exploration as a result of its significant impact on patient outcomes, care quality, nurse satisfaction, nurse retention, and healthcare organizations’ financial viability by primary studies [4–6].

During the pandemic, nurses were reported to work with poor practice conditions, including lack of personal protective equipment, high risk of infection, heavy workload, limited participation in decisions, and lack of tests to diagnose the disease among professionals and patients, as many researchers reported [7–9].

Therefore, given the importance of a healthy work environment for patients, nurses, and organizations, researchers and professional organizations have emphasized promoting nurse work environments [7, 9–11]**.** The World Health Organization (2020) called for urgent investment by improving work conditions [12]. The National Health Commission of China released a notice that required local health authorities and medical institutions to raise income and improve nurses’ practice environment [13].

The COVID-19 pandemic has lasted more than 2 years and has affected the nursing workforce, nursing education, healthcare delivery, policy and legislative issues, and social issues [14], which might also potentially affect the nursing practice environment. With the pandemic lasting and recurring in China, the pandemic prevention and control was normalized, and public hospitals are facing the dual pressure of pandemic prevention and control and resumption of business [15]. The measures of pandemic prevention and control were integrated into the healthcare workers’ daily work, including the management of entering and leaving the hospital the ward and environmental monitoring of the ward. The changes in work content increased the burden on human resources and improved the requirements for hospital service s[16]. However, the pandemic decreased the volume of business and surgery, which took challenges to hospital finance [16]. On the other hand, the pandemic situation was driving changes in the form of services. The incentive policy, practical experience, and the cultivation of people’s medical treatment habits during the pandemic brought a novel opportunity to develop internet-based health care and digital therapeutics [17]. Changes also occurred in the portrayals of healthcare workers in the media and public opinion environment. Since the pandemic, nurses were portrayed by the media as heroic, warm-hearted, and having a strong sense of professional morality [18]. Hospital service quality is given higher expectations and requirements.

The challenges and opportunities brought by COVID-19 and the changes in the overall situation might lead to some changes in the nursing practice environment, but these changes are still unknown. Studies have highlighted the necessity of continuously improving nursing practice environments [7, 19]. There has been a lack of research on what has happened to the nursing environment and how to improve it since the pandemic, especially from the nurses’ perspective. Thus, the present study aims to (1) evaluate nurses’ perceptions of the nursing practice environment since the pandemic and (2) investigate nurses’ views on the changes in the nursing practice environment brought by the COVID-19 pandemic and how to improve the nursing practice environment.

## Methods

### Study design, setting, and participants

This study adopted a cross-sectional study design, with quantitative and qualitative data collection at the same time to explore more details about the nursing practice environment. Convenience sampling was used to recruit nurses from seven tertiary hospitals in Sichuan Province, China. The inclusion criteria were clinical nurses working at least 1 year and volunteering to participate in this study.

The sample size was calculated according to the Kendall sample estimation method [20], which should be 10 ~ 20 times the number of variables. There were seventeen variables in this study. Consequently, the estimated sample size was more than 408, with the consideration of 20% efficiency.

The Strengthening the Reporting of Observational Studies in Epidemiology (STROBE) checklist for cross-sectional studies, the Checklist for Reporting Results of Internet E-Surveys (CHERRIES) [21], and the Mixed Methods Appraisal Tool (MMAT) [22] were applied to ensure the quality of reporting.

### Instruments

The self-administered questionnaire consisted of three parts:

Part A: Demographic and occupational information collected from participants included age, working seniority, monthly frequency of night shifts, monthly frequency of overtime, gender, marital status, education level, department, professional title, monthly income, the experience of caring for COVID-19 patients, and the experience of working related to COVID-19 prevention and control.

Part B: The Chinese version of the Practice Environment Scale-Nursing Work Index (PES-NWI) was used to assess the nursing practice environment. It was a widely used scale developed by Lake [3] and revised by Wang Li [23], which has good reliability with a Cronbach’s α of 0.91. The scale consisted of five dimensions and thirty-one items: (a) nurse–physician relations (3 items); (b) nurse manager ability, leadership, and support of nurses (5 items); (c) nursing foundations for quality of care (10 items); (d) nurse participation in hospital affairs (9 items); and (f) staffing and resource adequacy (4 items). It used a 4-point Likert scale, ranging between 1 (strongly disagree) and 4 (strongly agree). Values above 2.5 indicate agreement, and values below 2.5 indicate disagreement. Higher scores indicate more agreement that the items are present in the current job situation.

Part C: At the end of the questionnaire, two open-ended questions were set to capture richer information on the views of nurses about the impact of COVID-19: (a) “How do you think the COVID-19 has affected the nursing practice environment?” (Q1); (b) “As a nurse, how do you think to improve the nursing practice environment?” (Q2).

### Data collection

Data collection was conducted from June to July 2021. First, the questionnaire was uploaded to Wenjuanxing (https://www.wjx.cn), an online system widely used in academic studies in China. Then, the questionnaire link was sent to the nursing departments of seven tertiary hospitals through WeChat or QQ. The survey was open to each visitor of a site. An uncompleted questionnaire could not be submitted according to the setting of the link. And the questionnaire cannot be changed once it has been submitted. To prevent duplicating responses, we accepted only one questionnaire per person by tracking internet protocol addresses. Two research assistants (Peng WX and Zhu LL) downloaded and checked the data.

### Statistical analysis and content analysis

Frequency distributions were adopted to describe categorical variables of demographic and occupational characteristics. Means with standard deviations (SD) were used to describe age, working seniority, monthly frequency of night shifts, monthly frequency of overtime, and scores of PES-NWI. A t-test was carried out to compare the PES-NWI scores with the norm of Sichuan Province in 2010 in China [24]. Statistical significance was set at *p* < 0.05 (two-tailed). All statistical analyses were conducted with SPSS 26.0.

A deductive and in parts inductive unconstrained matrix analysis of the data collected from two open-ended questions was conducted according to the approach proposed by Elo [25] and Hansson [26] to make a corresponding comparison with the results of quantitative results. Two investigators (Cheng JX and Zuo QT) first read the text several times and identified relevant passages and sentences. Then, an analysis matrix was developed based on the PES, which resulted in some generic categories, of which some were coherent with the PES, but others were diverse. The investigators deconstructed the text, highlighted the words that captured key points, and then coded and grouped the content. The two investigators performed the analysis dependently to ensure trustworthiness and credibility, and any disagreements were resolved by discussion. The frequency distributions of the codes described the results of the content analysis.

### Ethical considerations

This study was approved by the Biomedical Ethics Committee, West China Hospital, Sichuan University (No. 2021 (612)). All participants were told the purpose and procedure of the research on the first page of the questionnaire and gave their informed consent by checking the confirm button after review. All of them were informed that participation was voluntary, their personal and institutional information would be kept confidential, and they could withdraw at any time during the survey.

## Result

### Participant characteristics

This study involved 470 clinical nurses and screened 460 valid responses after excluding nurses without consent or working less than 1 year, with a response rate of 97.8%. As illustrated in Table 1, the mean age of the participants was 32.25 years (SD = 6.98), with 90.1% being female and 66% being married. A total of 86.5% of the nurses had a bachelor’s degree or higher. In terms of occupational characteristics, more than half of the participants (59.8%) worked in the medical ward and surgery ward, and 71.7% had a junior professional title (nurse or senior nurse). The mean of working seniority was 10.30 years, and approximately half of the participants (54.1%) had a monthly income of over 9000 yuan, approximately 1400 dollars. Nurses worked an average of 5.40 (SD = 3.69) night shifts and worked overtime 3.94 (SD = 5.19) a month.

**Table 1:** Demographic and occupational Characteristics of Participants (*n* = 460)

Variable		Mean	SD
Age, years		32.25	6.98
Working seniority, years		10.30	7.97
Monthly frequency of night shifts		5.40	3.69
Monthly frequency of overtime		3.94	5.19
	Categories	Frequency	%
Gender	Male	42	9.1
	Female	418	90.1
Marital status	Married	304	66.0
	Unmarried	147	32.0
	Divorced or widowed	9	2.0
Education level	Junior college or below	62	13.5
	Bachelor	357	77.6
	Postgraduate	41	8.9
Department	Medical department	162	35.2
	Surgical department	35.2	24.6
	Pediatric department	113	18.7
	Emergency department	24.6	7.8
	Intensive Care Unit	86	8.3
	Other ^a^	25	5.4
Professional title	Junior title (nurse)	61	14.1
	Junior title (senior nurse)	249	57.6
	Intermediate title (Supervisor nurse)	108	25.0
	Senior title (Deputy chief nurse or Chief nurse)	14	3.3
Monthly income (RMB, yuan)	≤5000 (Approximately 780 dollars)	20	4.3
	5001 ~ 7000 (Approximately 780 ~ 1100 dollars)	86	18.7
	7001 ~ 9000(Approximately 1100 ~ 1400)	105	22.8
	≥9000 (Approximately 1400 dollars)	249	54.1
The experience of caring for Covid-19 patients	Yes	31	6.7
	No	429	93.3
The experience of working related to Covid-19 prevention and control	Yes	209	45.4
	No	251	54.6

^a^ Including obstetrics- gynecology department, operation room, and outpatient department

### The nursing practice environment

As demonstrated in Table 2, the mean scores of the PES-NWI and its five dimensions of nurse participation in hospital affairs, nursing foundations for quality of care, nurse manager leadership and support of nurses, staffing and resource adequacy, and nurse-physician relations were 3.43 (SD = 0.57), 3.28 (SD = 0.74), 3.61 (SD = 0.50), 3.53 (SD = 0.62), 3.19 (SD = 0.79), and 3.44 (SD = 0.61) out of 4.00, respectively. Compared with the results of the study conducted by Liu in 2010 [24], the results in this study demonstrated a higher score for the total scale and dimensions, apart from the dimension of nurse-physician relations and nurse participation in hospital affairs.

**Table 2:** The mean score of items of the practice environment scale and comparison between this study and Liu

Variable	This study (Mean (SD))	Norms (Mean (SD))	t	*P*
**Practice environment**	3.43 (0.57)	–	–	–
Nurse participation in hospital affairs	3.28(0.74)	3.40(0.97)	−2.382	0.017*
Nursing foundations for quality of care	3.61(0.50)	3.26(0.88)	8.011	< 0.001*
Nurse manager leadership, and support of nurses	3.53(0.62)	3.33(0.92)	4.278	< 0.001*
Staffing and resource adequacy	3.19(0.79)	3.06(1.05)	2.390	0.017*
Nurse–physician relations	3.44(0.61)	3.42(0.95)	0.489	0.677

*P < 0.05

### Content analysis

Excluding the responses giving irrelevant answers or without informed consent, 297 nurses provided 357 points of view for question 1, and 192 nurses provided 218 points of view for question 2. The frequency distributions of the major themes and their related codes were reported in Tables 3 and 4, respectively.

**Table 3:** Themes and Codes Emerging from Content Analysis of Nurses’ View of the Impact of COVID-19 on the Nursing Practice Environment (*N* = 357)

Theme	Code	Frequency (%)
Nurses’ perception of the impact		
	No impact	26(7.3)
	Positive impact	13(3.6)
	Have no idea	10(2.8)
	Negative impact	10(2.8)
Nurse participation in hospital affairs		
	More attention/recognition on nursing	42(11.8)
	Less opportunities for advancement momentarily	2(0.6)
Nursing foundations for quality of care		
	Improvement of nurse’s professionalism and skill	58(16.2)
	More stringent quality assurance program.	29(8.1)
	Higher standards of nursing care	17(4.8)
	Development of online service	4(1.1)
Staffing and resource adequacy		
	Heavier workload	56(15.7)
	Being short-staffed	15(4.2)
	Improvement on staffing and resource distribution	8(2.2)
Nurse–physician relations		
	Closer communication and cooperation	36(10.0)
	Enhanced team spirit	4(1.0)
Other		
	More difficulties in ward management	15(4.2)
	Better ward environment	6(1.7)
	Higher occupational risk	6(1.7)

**Table 4:** Themes and Codes Emerging from Content Analysis of nurses’ views of how to improve the nursing practice environment (*N* = 218)

Theme	Code	Frequency (%)
Nurse participation in hospital affairs	–	
	Being more recognized/respected	34(15.8)
	More opportunities for advancement of nursing personnel	5(2.3)
	Listening and responding to nurses’ concerns	3(1.4)
	Clinical ladder opportunity	2(0.9)
Nursing foundations for quality of care	–	
	More concise and standard work model	27(12.4)
	Professional development	13(6.0)
	Less formalism	7(3.2)
	Nurse’s professionalism and skill	7(3.2)
Nurse manager ability, leadership, and support of nurses		
	Human-based management	6(2.8)
	The leadership of nursing manager	2(0.9)
Staffing and resource adequacy		
	More staff	26(11.9)
	Support services	10(4.6)
	Lighter workload	13(6.0)
Nurse–physician relations		
	Increased collaboration between physicians and nurses	7(3.2)
Other		
	Comfort and safety of ward environment	40(18.3)
	A better deal	16(7.3)

### Nurses’ perception of the impact

Nurses expressed their intuitive feelings about the impact of COVID-19 on the nursing practice environment with wide variation. Most of them had complex feelings, *stating that “The changes only last during the pandemic, and after that, everything goes on as it is*. *-registered nurse (RN) 465*” and *“Impact of the pandemic was two-sided, presenting huge challenges as well as substantial opportunities. -RN85”.*

### Nurse participation in hospital affairs

Most statements related to the impact of the pandemic on nurse participation in hospital affairs were positive: *“Nurses played a vital role in pandemic prevention and control, which resulted in improved decision-making and a stronger voice. -RN171”.*

Regarding the improvements in nurses’ participation, more expectations in opportunities for advancement and a clinical ladder were expressed compared with strengthening their voices. They stated, *“I hope that the hospital could offer more opportunities for learning and further education. – RN233”* and *“It is difficult for us to promote. – RN49”.*

### Nursing foundations for quality of care

Most of the comments about the impact of the pandemic on nursing foundations for quality of care focused on nurses’ professionalism and skill and quality assurance programs. Representative statements included: *“In the face of the pandemic, I learned to be firm and good at self-regulation. -RN62”* and *“Each link of management is much more improved, but makes our work more complicated and overloaded. -RN273”.*

Some nurses thought the pandemic presented higher standards of nursing care and a better ward environment, as in these examples: *“Outbreak control places higher demands on nurses, both for professional skill and psychological resilience. -RN47”* and *“We restrict the person to enter the ward, which makes the ward environment quieter. -RN38”.*

Challenges brought by the pandemic also existed, including increasing difficulties in ward management and higher occupational risk: “*Some caregivers were unwilling to cooperate with anti-pandemic work. -RN447”* and “*Nurses are at risk of being infected. -RN9”.*

The suggestions about the improvement of nursing foundations for quality of care were focused on comfort and safety of the practice environment. Representative statements were *“Narrow resting and office spaces lead to poor experience of work. -RN462”* and *“Ward, especially emergency ward, need more independent and wide space. -RN156”.*

Nurses expected a more concise and standard work model so that clinical nurses have more energy to take care of patients rather than paperwork. They stated that *“The work content of nurses is very tedious, and we have no time to communicate with patients.* -RN47*”* and *“It is hoped that clinical efforts will focus on caring for patients, reducing paperwork, and other non-nursing related tasks. -RN99”.*

### Nurse manager ability, leadership, and support of nurses

Nurses did not comment on changes in nursing managers during the pandemic but put forward their expectations towards human-based management and improvements in nursing managers’ abilities. The representative statement was *“I hope managers pay more attention to the organizational culture construction and protection of nurses’ rights and interests. -RN201”.*

### Staffing and resource adequacy

Many nurses expressed their views that the anti-pandemic work contributed to a heavier workload and serious staff shortages. Representative quotations include the following: “*In the postepidemic period, the prevention of nosocomial infection is a long-term process, with more burden on nurses and shortage of manpower. -RN354”.*

Thus, the expectations of increased nursing staff and support services are prominent and strong. As a nurse said, “*More clinical nurses are needed to safeguard the quality of care. -RN411”* In addition, many nurses emphasized the importance of being respected and a better deal on reducing the turnover rate.

### Nurse–physician relations

The positive impact of anti-pandemic therapy on team spirit seemed evident, not only between nurses but also between physicians and nurses. As these nurses stated*, “Cooperation between healthcare professionals was closer. -RN240”* and *“More cohesive. -RN370”* Their suggestions about nurse–physician relations were increasing collaboration between physicians and nurses.

## Discussion

This study focused on the nursing practice environment, which is an important issue during the COVID-19 pandemic, and there has been limited research concerning this topic. The strengths of both quantitative and qualitative approaches were utilized to assess nurses’ perceptions and views. The quantitative and content analysis results were integrated to make more nuanced interpretations of the changes in the nursing practice environment since the COVID-19 pandemic.

It was revealed that nurses had positive perceptions of the nursing practice environment in this study. For further confirmation, we compared the results of the practice environment to normative data in 2010 [24] collected by using the PES-NWI in Sichuan Province before the COVID-19 pandemic, the results of which support the conclusion that the COVID-19 pandemic was associated with the improvement of the nursing practice environment. However, the results should be treated with reserve due to the differences in the characteristics of participants.

The age, working seniority, and education level of nurses in this study and the proportion of male nurses were higher than those in Liu’s study [24]. The recruitment of nursing managers in this study might partly explain the differences, including the older age and longer working seniority, which significantly impacted nurses’ perceptions of the practice environment [27]. This study aimed to explore the altitude and views of nursing managers with consideration of their important roles in the construction and improvement of the nursing practice environment. The more significant proportion of male nurses might benefit from the huge demand for male nurses and the change in traditional views. The changes in the structure of nurses’ education levels might be accounted for by the rapid development of Chinese nurse education in the past. The increased proportion of male nurses and highly educated nurses is beneficial for improvements in the structure of nursing talent but is related to a lower evaluation of the nursing practice environment [28, 29].

In addition to Liu’s study [24], the PES has been broadly adopted in other studies in varied participants and regions in China [30–34], which resulted in different scores but still lower scores than our findings. Meanwhile, many of these studies illustrated that the dimensions of staffing and resource adequacy and nurse participation in hospital affairs received the poorest score [30–34], which was also confirmed in our study. The score of nurse participation in hospital affairs in Liu’s study was relatively high, which might be the reason why the result of the comparison was not significant, although the qualitative data showed a positive response. The staffing dilemmas in nursing, an existing worldwide health problem, have intensified since the onset of COVID-19 [35] and might be constant due to the normalization management of the COVID-19 pandemic for some time [36].

The most significant change in the practice environment occurs in the dimension of nursing foundations for quality of care. One possible explanation is that this dimension, regarding quality assurance programs, service requirements, and nursing concepts, is most easily controlled by organizations under the cultural background of collectivism in China. On the other hand, more stringent procedures and excellent clinical competence were urgent requirements for nurses in the pandemic situation.

Nurses’ statements about leaders’ abilities were few, while quantitative analysis showed that changes in leaders were positive. An earlier study also revealed that the COVID-19 pandemic does not weaken nurses’ and managers’ leadership, although the pandemic has impacted their work and health, including communicating and implementing policy changes, surge staffing, staff well-being, access to personal protective equipment, staff retention, furloughs, and layoffs [37]. One possible explanation they gave was clinical nurses’ and nurse leaders’ perseverance and responsiveness despite a crisis [38]. The leadership of nursing managers is essential to work environments and outcomes both during and after the pandemic, which are times of crisis and significant change [39]. Now is the time for nurse leaders and active participants with other key decision-makers to be increasingly visible and to offer our creativity [40]. Under the situation of staff shortages and heavy workloads, the human-based management concept is expected and inspirational.

The change in physician–nurse collaboration was also encouraging; although the comparison result was not significant, the positive responses from nurses confirmed it. Maintaining the relationship between nurses and doctors under the principle of equality, respect, and win-win cooperation has been a mutual recognition to give full play to the ability of nurses in the epidemic prevention and control of major infectious diseases [41]. However, this closer cooperation established in anti-pandemic work may be temporary, which is also what most participants worried about. If we want to maintain this positive change, it is essential for nurses to improve their professional competence, voice, and social status.

Although some positive changes in the nursing practice environment were found, more long-term efforts are still needed to solve obstinately and ignored problems from the perspective of nurses. The most mentioned demand was a good ward environment, including ward facilities and staff rest environments. The impact of the physical environment on human health is widely known, so a safe and comfortable environment was built to facilitate the recovery of patients. However, nurses’ demands were often ignored due to the enormous medical demands, the concentration of medical resources, and limited space in hospitals. On the other hand, the definitions and measurements of the nursing practice environment focused more on the internal social environment of the organization. Thus, while this might sound like a bit of concern, it is nevertheless a crucial problem that managers need more attention to and investment.

In addition to the nagging problem of staff shortages and low recognition/low participation mentioned earlier, another concern is the work model. Nurses in this study had a good evaluation of nursing foundations for quality of care with the quality assurance program and service requirement involved. Nevertheless, although they highlight the importance of these standardization processes, they expressed negative impacts such as increased workload and staff stress. This agrees with a study conducted in Brazil during the COVID-19 pandemic [7]. Nurses opposed tedious work sessions and paperwork and looked forward to more concise and efficient work models that allow nurses to invest more energy into professional services. As advocated by Lake [3], a professional model that emphasizes goal-centered, individual qualifications, and collegial control systems operating within the professional staff is preferable to a bureaucratic mode that emphasizes task-centered control exercised through hierarchical authority and formal rule enforcement.

This study has several limitations. First, as mentioned earlier, the results of the quantitative analysis should be taken with caution due to the differences in demographic characteristics. Second, convenient sampling might reduce the representativeness of the sample. Third, the information provided by the qualitative data collected online may be insufficient, although we thought it might reflect nurses’ core views. Despite these limitations, the findings of this study provide some enlightenment for the improvement of the nursing practice environment during the pandemic.

## Conclusion

The COVID-19 pandemic is not just a time of crisis but an opportunity for achieving a better nursing practice environment. Some good changes in the nursing practice environment occurred, including improvement of nurse’s professionalism and skill, more attention/recognition on nursing, and closer communication and cooperation. More efforts are needed to solve the problems of staff shortages and low participation. Given the particular period found, nursing managers and hospital leaders were suggested to listen to nurses’ concerns about the practice environment and value this suitable opportunity to change and improve to achieve better health services and coping ability to deal with emergency events. Improving the ward environment and taking a professional model instead of sticking to the tedious process might be worthwhile.

## Data Availability

The datasets used and/or analysed during the current study are available from the corresponding author on reasonable request.
